# University makes me angry: Investigating stimulus-response (S-R) and cognitive-mediation (C-M) emotion beliefs in undergraduate students

**DOI:** 10.1371/journal.pone.0294777

**Published:** 2024-02-14

**Authors:** Martin J. Turner, Daniel Boatwright, Andrew L. Evans, Gulcan Garip, Charlotte Chandler, Nanaki J. Chadha, Andrew G. Wood

**Affiliations:** 1 Department of Psychology, Manchester Metropolitan University, Manchester, United Kingdom; 2 Sport and Exercise, Staffordshire University, Staffordshire, United Kingdom; 3 School of Health and Society, The University of Salford, Manchester, United Kingdom; 4 College of Health, Psychology and Social Care, University of Derby, Derbyshire, United Kingdom; 5 College of Science and Engineering, University of Derby, Derbyshire, United Kingdom; Universidad Central de Chile, CHILE

## Abstract

Emotion regulation through cognitive reappraisal is well-studied, but less so are the predispositional and superordinate beliefs that influence reappraisal. Recently, researchers developed the cognitive mediation beliefs questionnaire (CMBQ), which measures two emotion beliefs, namely stimulus-response (S-R) generation beliefs and cognitive mediation (C-M) change beliefs. In working populations S-R generation beliefs are inversely related to cognitive reappraisal tendencies and positive mental health, and positively related to emotion reactivity. C-M change beliefs are positively related to cognitive reappraisal tendencies, and inversely related to emotion reactivity and positive mental health. As yet, there is no evidence for the validity of the CMBQ within student samples, or for the associations between its subscales and cognitive reappraisal, emotion reactivity, and positive mental health. Therefore, in the present study the CMBQ is tested for factorial, convergent (associations with cognitive reappraisal), and concurrent (associations with emotion reactivity and positive mental health) validity in a cohort of 621 undergraduate students in the United Kingdom (U.K.). Results indicate support for the factorial and convergent validity of the CMBQ, with mixed evidence for the concurrent validity of the CMBQ. A CM-SR discrepancy score appeared to provide a promising variable when associated with emotion reactivity and positive mental health. The findings are discussed in terms of practical and research implications of the findings.

## Introduction

University students face mounting pressures both within and outside of the academic milieu, and there is evidence that being a student may become a stressful experience [1]. Not only are there marked pressures for students to achieve the best grade possible at university, they also *pressure themselves* to fulfil their potential. Amidst these performance pressures, university students today face a volatile political landscape (i.e., Brexit), an interpersonal setting in which social media has become a dominant and pervasive force, and the financial burden of student fees and a cost-of-living crisis. On top of these factors, many university students are living away from home for the first time and are navigating independent living in isolation of family. The stressful context that students occupy is being reflected in rising mental health difficulties. A large-scale study (University Student Mental Health Survey, 2020) reported that 42.3% of students had a serious personal, emotional, behavioural or mental health problem for which they needed professional help, and 26.6% of students had received a mental health diagnosis. Another report (A Degree Of Uncertainty: Looking At Student Wellbeing, 2020) indicated that 37% of students believed their state of mental wellbeing deteriorated since they started studying, and 64% of students reported that studies and university lifestyle negatively impacted their state of mental wellbeing. Mark Crawford, a postgraduate student union officer, writing for Red Pepper, stated that “Driving our universities to act like businesses doesn’t just cannibalise the joy of learning and the social utility of research and teaching; it also makes us ill” [2].

One factor that can increase the risk, and exacerbate the symptoms, of mental illness, is difficulty in emotion regulation [3]. Emotion regulation refers to attempts to influence one’s emotions [4], and successful emotion regulation is beneficial for various psychological and physical health outcomes [e.g., 5, 6] and, fortunately, there are many strategies one can employ to regulate emotion. These strategies have been conceptualised in Gross’ [7] process model of emotion regulation, comprising of strategies such as situation selection, situation modification, attentional deployment, cognitive change, and response modulation. However, not all emotion regulation strategies are equal. Cognitive reappraisal (or cognitive change) is demonstrably one of the most effective [8], and well-studied [9] emotion regulation strategies. Cognitive reappraisal is the modification of one’s appraisal of a situation to affect its emotional impact [4] and has been associated with many adaptive outcomes, both psychophysiologically [e.g., 10], and neurologically [e.g., 11]. The efficacy of cognitive reappraisal for successful emotion regulation is not just espoused by emotion scientists but is also the backbone of second wave cognitive behavioural therapies (CBTs), in which cognitive mediation is a key axiomatic principle [12]. That is, our thoughts about events shape our emotional reactions [13]. Thus, in many CBTs, patients are encouraged to understand the role of and to modify their maladaptive cognitions as a way to regulate emotion. The idea that cognitions mediate between stimuli and emotion is captured in theory [e.g., 14], and scientific evidence [see 15], and underpins second wave CBTs such as cognitive therapy [CT; 16] and rational emotive behaviour therapy [REBT; 17] where cognitive change is paramount.

Research in university students is equivocal with regards to emotion regulation development, with some research indicating no change in cognitive reappraisal capacity among students during their studies [e.g., 18, 19], and some research suggesting an increased use of maladaptive emotion regulation [suppression: 20], whilst other research reports decreased suppression and increased reappraisal [21]. This equivocality is troubling amidst the evidence that increases in maladaptive emotion regulation strategies occur with age [22], and the findings that greater tendencies to utilise adaptive emotion regulation strategies (cognitive reappraisal) is related to better personal and social wellbeing outcomes [19], reduced suicidal behavior [23], and better psychological and general health [24]. Therefore, research that aims to understand the predetermining factors that could predict greater engagement in adaptive emotion regulation strategies, such as cognitive reappraisal, in university (college) student populations is needed.

Given the effectiveness of cognitive reappraisal for emotion regulation, an understanding of the factors that could predetermine reappraisal attempts is important to study. That is, if we know the preceding factors that make reappraisal attempts more likely, then we can seek to influence those preceding factors with a view to helping students regulate emotion adaptively. One potential preceding or concomitant concept that has emerged in recent emotion regulation literature is “emotion beliefs” [15, p. 74], considered to be beliefs about emotion and emotion regulation. Individual differences in what people believe about emotion and emotion regulation have meaningful consequences for emotion regulation [25, 26]. In other words, is it proposed that what we believe about our emotions can influence our attempts to regulate emotions. For example, the belief that emotion is malleable leads to higher emotional regulation capacity, that predicts better well-being, interpersonal functioning, and mental health [27–29]. However, research in the field of emotion beliefs is still in its infancy [e.g., 30], although it is growing [31].

Amidst the burgeoning research into emotion beliefs, recently Turner et al. [32] conceptualised two superordinate emotion beliefs, measured using the cognitive-mediation beliefs questionnaire (CMBQ), that show promise in initial findings. These emotion beliefs are:

1. Stimulus-Response (S-R) generation beliefs (the belief that emotions are caused by events)
2. Cognitive Mediation (C-M) change beliefs (the belief that changes in cognition lead to emotion change).

To expand, S-R generation beliefs reflect the idea that emotion is solely caused by external situational events, and C-M change beliefs reflect the idea that emotion can be modified through cognitive reappraisal (or cognitive change). Initial findings concerning S-R generation and C-M change beliefs [32] indicate that greater C-M change beliefs and lower S-R generation beliefs are related to higher cognitive reappraisal tendencies (adaptive emotion regulation), greater ability to control thoughts, more positive mental health outcomes, and lower emotion reactivity (less persistence, sensitivity, and intensity of emotion). In brief, one’s beliefs about emotion can indicate the extent to which one engages in particular emotion regulation attempts, such as cognitive reappraisal. As such, an individual with the belief that their emotions are caused solely by external events (S-R generation), is less likely to engage in cognitively driven emotion regulation strategies, such as cognitive reappraisal. This might be because an individual with high S-R generation beliefs may not recognise the role of cognitions in emotion aetiology and thus is not likely to employ a distinctly cognitive emotion modification strategy. The colloquial articulation of S-R generation beliefs can be witnessed easily in daily interactions with one another; “it makes me nervous”, “they made me angry”, “it made me feel really guilty”. Technically, these statements are not accurate–an external event cannot singlehandedly *make* us feel anything, rather, it is the meaning we ascribe to events that shapes our emotion [33], not events alone.

In contrast, an individual with the belief that emotions can be cognitively mediated (C-M change), is more likely to engage in cognitive reappraisal [32, 34]. This is important because of the support for cognitive reappraisal as an effective strategy for emotion regulation [e.g., 8], and thus, emotion beliefs that could indicate reappraisal likelihood (i.e., less S-R and more C-M) might tell us more about how we can encourage adaptive emotion regulation. Indeed, in the second wave CBTs it is typical to help patients understand the important role of cognition in their emotions, and encourage them to take charge of their cognitions in order to enable greater emotion regulation [35, 36]. An understanding of S-R generation and C-M change beliefs can help us reflect on Mark Crawford’s [2] aforementioned statement that the business-like actions of universities “makes us ill” (S-R generation) and help us to understand the environment-individual transaction in student emotion reactivity and mental health difficulties.

The current paper concerns the utility and validity of S-R generation and C-M change emotion beliefs for university students studying in the United Kingdom (U.K.). There were two aims of the current paper. First, we aimed to test the factor structure (factorial validity) of the CMBQ [32], a self-report psychometric that measures S-R generation and C-M change beliefs, with a student cohort for the first time. The CMBQ was initially developed within an occupational sample, and thus, prior to subsequent hypothesis testing, it was important to ensure that the measure was reliable in the student sample recruited for this study. Second, we aimed to examine the convergent and concurrent validity of the CMBQ by investigating the associations between S-R generation and C-M change beliefs, and cognitive reappraisal tendencies (convergent validity), and markers of emotion reactivity and positive mental health (concurrent validity). It is proposed in previous research [32, 34], and thus is hypothesised in the current study, that cognitive reappraisal tendencies should be inversely related to S-R generation beliefs, and positively related to C-M change beliefs, such that lower scores in S-R generation beliefs and higher scores in C-M change beliefs should be related to greater tendencies to apply cognitive reappraisal emotion regulation strategies. Also, in line with past research [32], it is hypothesised that greater S-R generation beliefs and lower C-M change beliefs will be related to higher (poorer) emotion reactivity and lower (poorer) positive mental health. In sum, it was hypothesised that the CMBQ would demonstrate factorial, convergent, and concurrent validity in an undergraduate student sample.

## Materials and methods

### Participants

In order to minimize errors and maximize the accuracy and generalizability of population estimates in scale validity and reliability testing, an a priori participant:item ratio of 10:1 was considered [37, 38], alongside guidelines that between 500 (very good) and 1000 (excellent) participants is suitable [39]. Thus, six hundred and twenty-one students participated in the present study (*M*age = 23.64; *SD*age = 8.25; female = 304, male = 272, did not disclose = 45; Asian = 49, Black = 26, Mixed = 14, White = 484, did not disclose = 48; single = 398, married = 55, divorced = 5, in a relationship = 29, did not disclose = 134). Participants were recruited from four universities in the United Kingdom (U.K.) via convenience and snowball sampling between November 2019 and March 2021 by inviting prospective participants to take part via course virtual learning environments and in physically in class, and then asking students to circulate the information to fellow students in their year. Participants were mostly full-time students (fulltime = 598, part-time = 23) in their first year of undergraduate study (1^st^ year undergraduate = 274, 2^nd^ year undergraduate = 162, 3^rd^ year undergraduate = 87, postgraduate = 88, doctoral = 9, did not disclose = 1). Questionnaires were completed either online using Qualtrics (online survey provider), or physically in person using paper surveys. The questionnaires took no longer than 15-minutes to complete.

### Design

We adopted a cross-sectional single timepoint study design, allowing us to test the hypotheses using confirmatory factor analysis, bivariate correlations, and multiple linear hierarchical regression.

### Measures

#### Cognitive mediation beliefs

The 15-item CMBQ [32] (S-R generation = 8 items, C-M change = 7 items) was scored on a 1 (*strongly disagree*) to 5 (*strongly agree*) Likert-scale (see Table 1 for the CMBQ items). Cronbach’s *α* for the current sample was .88 for S-R generation, and .82 for C-M change. Prior to distributing the questionnaire to prospective participants, we engaged ten undergraduate students (female = 7, male = 3; White = 8, Asian = 2; age range 19–24) in a small pilot study of the CMBQ to assess the face validity [e.g., 40] of the CMBQ within the undergraduate student population. Pilot participants completed the CMBQ online and were asked to indicate what they thought of the CMBQ, whether they could discern C-M change items from S-R generation items, and whether the items were readable or not. Specifically, participants were given a definition of C-M change and S-R generation beliefs, and then asked to read each item of the CMBQ thoroughly. They were asked to indicate which of either C-M change or S-R generation beliefs each item assessed, and then to score each item between 1 and 10 on item accuracy (how accurately the item captures either C-M change or S-R generation beliefs) and clarity (how clearly the item is worded) with higher scores indicator greater accuracy and clarity respectively. Students were also invited to write down any comments they had about each item as to its quality. All participants correctly identified which item belonged to which CMBQ subscale, all items were deemed to be accurate (M = 8.70, SD = .54, range = 8.00–9.30) and clear (M = 8.94, SD = .47, range = 8.20–9.50). Students remarked that the items were easy to read, but some were repetitive, and eight students indicated that C-M change items were more desirable, one student indicated that S-R generation items were more desirable, and one student suggested a mix of C-M change and S-R generation was desirable. In all, the pilot indicated that the CMBQ demonstrated face validity, and thus we did not alter any items. The notion that items are repetitive is a feature of psychometric instruments and one that is important for internal validity. Therefore, we commenced participant recruitment forthrightly.

**Table 1:** Item properties, internal consistency, inter-item correlations, and descriptives, of the 15-item CMBQ.

					Inter-item correlation	
	*β*	*R* ^2^	*α*	M(SD)	Range	M(SD)
**S-R generation**			.88			
How I feel is completely dictated by the things that happen to me in my life.	.43	.19		3.12(1.00)	.281-.474	.351(.062)
My feelings are entirely determined by peoples’ actions towards me.	.72	.52		3.04(1.02)	.359-.580	.496(.085)
My feelings are completely controlled by the situation I am in.	.75	.56		3.09(1.04)	.346-.595	.511(.088)
My emotions are entirely caused by what people do around me.	.76	.58		3.00(1.03)	.307-.634	.510(.119)
My emotions are caused entirely by others’ actions towards me.	.76	.58		2.95(1.07)	.281-.634	.511(.119)
My emotions are caused entirely by the things that happen to me.	.72	.52		3.20(1.00)	.328-.642	.498(.093)
What happens to me entirely dictates how I feel.	.79	.63		2.95(1.03)	.388-.571	.458(.060)
My emotions are completely dictated by what happens to me.	.58	.34				
**C-M change**			.82			
To change how I feel, my thoughts about the situation need to change.	.64	.40		3.53(.99)	.218-.492	.379(.094)
To change how I feel, I need to change what I think about things around me.	.52	.27		3.68(.78)	.282–391	.349(.038)
Thinking differently about the situation will change how I feel.	.63	.40		3.61(.86)	.380–477	.409(.036)
To change how I feel, I can change my thoughts about the situation.	.63	.39		3.67(.81)	.218-.521	.373(.100)
I can change my emotions by changing how I think about the situation.	.76	.58		3.49(.92)	.341-.521	.466(.066)
Because I can choose to think differently, I can choose to feel differently about the situation.	.63	.39		3.39(.98)	.282-.509	.396(.077)
To control my emotions, I need to change the way I think.	.66	.44		3.55(.92)	.327-.499	.420(.073)

#### Emotion regulation

The Emotion Regulation Questionnaire (ERQ) [41] is a 9-item [42] measure assessing the tendency to regulate emotions in two ways: (1) Cognitive Reappraisal and (2) Expressive Suppression. In the current, only reappraisal was measured due to its conceptual relevance to the CMBQ. Items were scored on a 7-point Likert scale from 1 (*strongly disagree*) to 7 (*strongly agree*). In the current sample, Cronbach’s *α* was .82.

#### Emotion reactivity

The Emotion Reactivity Scale (ERS) [43] is a 21-item measure emotion reactivity, that assesses emotion sensitivity, intensity, and persistence. For the current study we used the ERS total score (Cronbach’s *α* was .96), whereby higher scores indicate greater emotion reactivity.

#### Affective reactivity

The Affective Reactivity Index (ARI) [44] is a 6-item measure of chronic irritability with questions pertaining to anger threshold, anger frequency, and anger duration. In the current sample, Cronbach’s *α* was .86.

#### Positive mental health

The 9-item Positive Mental Health (PMH) scale [45] assesses emotional aspects of well-being via positive emotionality. Cronbach’s *α* was .90 in the current study.

### Data analysis

Data were screened for missing cases. Cases that were missing completely at random (Little’s MCAR *p* > .05) were replaced using the Expectation Maximization (EM) method. In all, 6 cases for CMBQ, 3 cases for ERQ, 42 cases for ERS, and 2 cases for PMH were MCAR and replaced. Data were also screened for outliers (standardized *z* values > 3.29), and outliers were Winsorized (*n* = 39 from 34,776 cases = .11%). Project data can be found in S1 File.

For main analyses, first, the 15-items of the CMBQ were subjected to CFA using SEM in AMOS version 25 [46], whereby a correlated two-factor model was tested (Table 1).

We subjected the CMBQ to CFA following guidelines for best practices, it is recommended that multiple factor analysis be performed within different populations to increase the factorial validity [37] previously the measure has only been tested in working populations [32], but not in student populations. Thus, we first sought to confirm the structure of the CMBQ in the student sample. The goodness of fit indices posited by Schermelleh-Engel et al. [47] were used to determine an acceptable fit. Specifically, goodness of fit was assessed using the χ^2^ statistic, the comparative fit index (CFI), the standardised root mean square residual (SRMR), and the root mean square error of approximation (RMSEA). Values close to .08 for the RMSEA and .08 for the SRMR are indicative of an acceptable model fit, as are values above .90 for the CFI [48, 49; also see 50]. The modification indices (MI) guidelines by Rossier et al. [51] were applied (< .20). Also, in the current study the covarying of subfactor item errors occurred where subfactor items possessed similarities in item content [52].

Second, in line with the original CMBQ research [32], we calculated Pearson’s correlation coefficients to examine the bivariate associations (between C-M and S-R beliefs, and cognitive reappraisal to assess the convergent validity of the CMBQ in a student sample.

Third, to assess the concurrent validity of the CMBQ in a student population, we conducted two sets of linear hierarchical multiple regression analyses. The first set were in line with the original CMBQ research [32] which regressed emotion reactivity (ERS and ARI) and positive mental health (PMH) onto C-M and S-R beliefs (step 2), whilst controlling for the effects of age, sex, study level (from 1 = undergraduate to 4 = post-graduate), and mode of study (full-time, and part-time) (step 1). The second set addressed a call by Turner et al. [32] to examine the effects of a CM-SR beliefs discrepancy score on emotion reactivity and positive mental health. That is, whilst C-M and S-R beliefs may have independent effects on emotion reactivity and positive mental health [32], it could be that the extent to which one reports C-M beliefs over and above S-R beliefs, and vice versa, is more indicative of emotion reactivity and positive mental health. Indeed, a person can have high C-M change beliefs *and* high S-R generation beliefs, and whilst inversely related, they are not necessarily orthogonal. We regressed emotion reactivity (ERS and ARI) and positive mental health onto a C-M and S-R discrepancy (CM-SR) score (step 2), whilst controlling for the effects of age, sex, study level, and mode of study (step 1). The CM-SR discrepancy scores were calculated by subtracting S-R beliefs scores from C-M beliefs scores, similar to the hedonic balance score derived from the Positive and Negative Affect Schedule (PANAS) [e.g., 53].

Finally, to explore potential differences in the S-R generation and C-M change scores between study levels, we conducted a 4 x 2 between-subjects MANCOVA, accounting for the effects of participant age as a covariate. There were four between-subjects factors, namely undergraduate level 1 (UG1), undergraduate level 2 (UG2), undergraduate level 3 (UG3), and post-graduate level (PG). For PG level we include doctoral participants because the low N of this population (N = 9) precluded its use as a separate group.

## Results

### CFA for CMBQ (factorial validity)

The 15-item two-factor model was a good fit, *χ*^2^ = 388.473, df = 86, *p* < .001, RMSEA = .075 (90% CI = .068–.083), CFI = .92, SRMR = .065. See Table 1 for factor loadings. C-M change and S-R generation were negatively related (-.23).

### C-M and S-R and cognitive reappraisal (convergent validity)

Pearson’s correlation coefficients revealed a positive association between C-M change scores and cognitive reappraisal (*r* = .36, p < .001), and a negative association between S-R generation scores and cognitive reappraisal (*r* = -.19, p < .001). In sum, greater C-M change and less S-R generation beliefs were related to greater cognitive reappraisal tendencies.

### Emotion reactivity and positive mental health onto C-M and S-R (concurrent validity)

For ERS scores, step 1 (demographic variables) explained a significant proportion of variance (*R*^2^Δ < .08, *p* < .001). In step 2 C-M change and S-R generation scores explained 11% of variance. In the final model, *F*(6,558) = 22.17, *p* < .001, sex was positively related to ERS scores (*β* = .24, *t* = 5.86, *p* < .001), as was study level (*β* = .10, *t* = 2.00, *p* = .047), and so to was S-R generation (*β* = .35, *t* = 8.77, *p* < .001). C-M change scores were not related to ERS scores (*β* = .07, *t* = 1.86, *p* = .063).

For ARI scores, step 1 explained a significant proportion of variance (*R*^2^Δ < .02, *p* = .014). In step 2 C-M change and S-R generation scores explained 7% of variance. In the final model, *F*(6,558) = 9.08, *p* < .001, S-R generation was positively related to ARI scores (*β* = .27, *t* = 6.35, *p* < .001). C-M change scores were not related to ARI scores (*β* = -.01, *t* = -.14, *p* = .89).

For PMH scores, step 1 explained a significant proportion of variance (*R*^2^Δ = .02, *p* = .027). In step 2 C-M change and S-R generation scores explained 3% of variance. In the final model, *F*(6,558) = 4.86, *p* < .001, sex was negatively related to PMH scores (*β* = -.01, *t* = -2.19, *p* = .029), and so too was year of study (*β* = -.12, *t* = -2.29, *p* = .022). S-R generation was negatively related to PMH scores (*β* = -.10, *t* = -2.31, *p* = .021), and C-M change scores were positively related to PMH scores (*β* = .14, *t* = 3.27, *p* = .001).

In sum, females, those in a higher level of study, and those reporting higher S-R generation reported greater emotion reactivity and lower positive mental health. In addition, those reporting higher C-M change reported higher positive mental health.

### Emotion reactivity and positive mental health onto CM-SR discrepancy (concurrent validity)

For ERS scores, step 1 explained a significant proportion of variance (*R*^2^Δ < .08, *p* < .001). In step 2 CM-SR discrepancy scores explained 5% of variance in ERS scores. In the final model, *F*(5,559) = 16.48, *p* < .001, sex was positively related to ERS scores (*β* = .27, *t* = 6.59, *p* < .001), but CM-SR discrepancy was negatively related to ERS scores (*β* = -.23, *t* = -5.57, *p* < .001).

For ARI scores, step 1 explained a significant proportion of variance (*R*^2^Δ < .02, *p* = .014). In step 2 CM-SR discrepancy scores explained 4% of variance. In the final model, *F*(5,559) = 7.96, *p* < .001, sex was positively related to ARI scores (*β* = .10, *t* = 2.42, *p* = .016), but CM-SR discrepancy was negatively related to ARI scores (*β* = -.22, *t* = -5.16, *p* < .001).

For PMH scores, step 1 explained a significant proportion of variance (*R*^2^Δ < .02, *p* = .027). In step 2 CM-SR discrepancy scores explained 3% of variance. In the final model, *F*(5,559) = 5.62, *p* < .001, sex was negatively related to PMH scores (*β* = -.09, *t* = -2.06, *p* = .039), and so too was year of study (*β* = -.12, *t* = -2.31, *p* = .021). CM-SR discrepancy was positively related to PMH scores (*β* = .18, *t* = 4.09, *p* < .001).

In sum, females, and those reporting lower CM-SR discrepancy scores (lower C-M change relative to higher S-R generation) reported greater emotion reactivity and lower positive mental health. Higher year of study was related to lower positive mental health.

### CMBQ scores between study levels

The 4 x 2 MANCOVA revealed a significant main effect for student level, Wilks Λ = .91, *F*(6, 1202) = 9.69, *p* < .001, ηp2 = .05. At the univariate level, S-R generation differed between groups, *F*(3,602) = 10.44, *p* < .001, ηp2 = .05, and so too did C-M change, *F*(3,602) = 9.46, *p* < .001, ηp2 = .05. For S-R generation, pairwise comparisons revealed that participants at PG level (*M* = 2.55, *SD* = .93) scored significantly lower (all *p* < .001) than UG1 (*M* = 3.12, *SD* = .66), UG2 (*M* = 3.16, *SD* = .72), and UG3 (*M* = 3.29, *SD* = .68) levels. For C-M change, pairwise comparisons revealed that participants at UG1 (*M* = 3.43, *SD* = .59) scored significantly lower (*p* < .001) than participants at UG2 (*M* = 3.72, *SD* = .57), that participants at UG2 scored significantly higher (*p* < .001) than participants at UG3 (*M* = 3.41, *SD* = .60), and that participants at UG3 scored significantly lower (*p* = .028) than participants at PG level (*M* = 3.82, *SD* = .66). As can be seen in Table 2, data indicate that the highest scores in C-M change and the lowest scores in S-R generation are reported by PG level students.

**Table 2:** Means and SDs for CMBQ data between study years.

	UG1	UG2	UG3	PG
	M (SD)	M (SD)	M (SD)	M (SD)
S-R generation	3.12 (.66)	3.16 (.72)	3.29 (.68)	2.55 (.93)
C-M change	3.43 (.59)	3.72 (.57)	3.41 (.60)	3.82 (.66)

*Notes*. UG = undergraduate, PG = post-graduate.

## Discussion

The chief purpose of the present study was to test the factor structure, and convergent and concurrent validity, of the CMBQ within a student cohort for the first time. The results confirmed the correlated two-factor structure of the 15-item CMBQ (factorial validity), offered support for its convergent validity, and indicated support for its concurrent validity, as hypothesised. Specifically, CFA indicated that the C-M change and S-R generation subscales offered a good fit to the data. Also, C-M change was positively related, whilst S-R generation was negatively related, to cognitive reappraisal. Further, greater S-R generation was associated with greater emotion reactivity and lower positive mental health, whilst greater C-M change was related to higher positive mental health but was not related to emotion reactivity. In addition, a CM-SR discrepancy score, whereby higher scores reflect greater C-M beliefs relative to S-R beliefs, was negatively related to emotion reactivity and positively related to positive mental health. Results are largely in line with previous research [26], and theory [54] concerning emotion beliefs, as well as previous findings specific to C-M and S-R beliefs [32]. However, full support could not be offered due to the equivocal findings regarding C-M change beliefs and emotion reactivity in the current sample.

The finding that C-M change beliefs were not related to markers of emotion reactivity could indicate that endorsing the beliefs that emotions can be altered by changing one’s thinking does not have implications for emotion reactivity. However, holding C-M change and/or S-R generation beliefs does not necessarily impact upon emotion reactivity directly. That is, Turner et al. [32] propose that holding high C-M change beliefs predisposes individuals to cognitive reappraisal attempts, thus it is through cognitive reappraisal that emotions are regulated. The positive relationship between C-M change beliefs and cognitive reappraisal tendencies found in the current study is indicative of this proposal. Holding high C-M change beliefs is perhaps unlikely to be beneficial for emotion regulation unless it leads to the enlistment of cognitive reappraisal. The same argument could be made for S-R generation beliefs, that although S-R generation beliefs were inversely associated with emotion reactivity, it could be argued that these effects can occur only through or via diminished attempts at cognitive reappraisal.

A possible process through which emotion beliefs might influence emotion reactivity and mental health might start with deeply held beliefs about emotion, which could predispose us to certain emotion regulation strategies, which then shape emotion reactivity. For example, one might hold beliefs that emotions are caused only by external events (high S-R generation) and that I cannot alter my emotions via cognitive change (low C-M change), which predisposes me to less attempts at cognitive restructuring in the face of stimuli, and resultant high emotion reactivity. To test these assumptions, one would need to adopt temporal and or experimental research methods to apply mediation analyses, for example, to determine the causal relationships between emotion beliefs and reactivity through cognitive reappraisal.

Also, we must consider that C-M change beliefs reflect beliefs about change, whereas S-R generation beliefs reflect aetiology. It is possible that the processes related to emotion *generation*, are separable from the processes that relate to emotion *management* [e.g., 55, 56]. It could be that high S-R generation beliefs are suggestive of perceptions of a bottom-up emotion generation process (i.e., elicitation of emotion by the presentation of a stimulus that is inherently emotional) [57], rather than a top-down process (i.e., elicitation of emotion by the activation of high-level appraisals) [58]. The differences in psychological and neural mechanisms for bottom-up vs. top-down emotion generation [e.g., 59] may have important consequences for emotion regulation attempts. Thus, strongly believing that emotions occur as a direct result of external stimuli (S-R generation) may discount cognitive reappraisal as an emotion regulation strategy, since the role of cognition in emotion per se is ignored. As such, a strong S-R generation belief may be more directly related to emotion reactivity compared to C-M change beliefs.

The above points are perhaps illustrated by the findings in the current study concerning CM-SR discrepancy scores, where higher scores reflect greater C-M change and lower S-R generation beliefs. When considered independently, C-M change and S-R generation beliefs have variable associations with emotion reactivity, as discussed. But when taken together as a relative index of CM-SR beliefs, more consistent associations with emotion reactivity were found. Thus, it is perhaps the interaction between C-M change and S-R generation beliefs that is important for emotion reactivity outcomes, rather than each subscale alone. Because C-M change and S-R generation beliefs appear not to be orthogonal (one can score highly in both), then we must account for the interaction between each belief when making predictions concerning emotion reactivity. For example, perhaps C-M change beliefs are only indicative of emotion reactivity when S-R generation beliefs are accounted for. This is a clear area for future research, and one that could be approached by applying temporal mediation analyses whereby S-R generation is assessed as mediator of the relationship between C-M change and emotion reactivity. This would allow us to make conclusions closer to cause-effect than is possible at present due to the cross-sectional nature of the designs utilised to examine S-R generation and C-M change beliefs.

In the present study, we separate emotion beliefs from cognitive reappraisal and emotion reactivity and mental health outcomes. It is possible that emotion generation and regulation are inseparable [25], however, treating them as separate for research is favourable [60]. Future research should more comprehensively examine the implications of C-M change and S-R generation beliefs on the process of emotion regulation, in line with the process model proposed by Gross [7]. We do not know at what point emotion beliefs influence emotion regulation, and we know little about how emotion beliefs may implicate other emotion regulation strategies, such as situation selection, situation modification, attentional deployment, and response modification. It could be implied that stronger S-R generation beliefs relative to C-M change beliefs might underpin situation selection and modification strategies, since the most viable approach for one who believes situations directly cause emotion is to change the causal agent. But this is conjecture and is in need of future research.

Alongside findings pertaining to the validity of the CMBQ, sex differences and study year effects also emerged in the present study. Specifically, female students reported emotion reactivity and lower positive mental health than males. This finding is partially in line with the original CMBQ validation study [32] in an occupation sample, in which females reported higher emotion reactivity scores than males, but no differences in positive mental health were found. It is not possible to categorically state why these sex differences emerged, however, sex has been found to be an important influencer of emotion regulation in past research [e.g., 61], possibly echoing the sex differences in the prevalence of psychopathologies that are characterised in part by deficits in emotion regulation [e.g., 62]. Sex differences in mental health have been well-established in past research. For example, Scott-Young et al. [63] found that female undergraduate students’ overall mental health deteriorated over the course of their degree programme whilst male undergraduate students’ overall mental health improved. Compared to males, female students have also reported higher overall levels of stress [e.g., 64], depression [e.g., 65], and anxiety [e.g., 65, 66]. Whilst our findings are consistent with extant literature, whether and to what extent sex is implicated in C-M change and S-R generation emotion beliefs remains to be fully explored.

The finding that participants in a higher year of study report worse positive mental health is enlightening and concerning. Data indicate increasing numbers of U.K. students reporting mental health problems [67], with recent data indicating high levels of depression and anxiety, with scores above the clinical cut off for over half of students sampled [68]. This finding suggests that, for the sample studied, as one progresses through academic study, mental health declines. However, we have to be careful with our conclusions here because we do not have longitudinal data that indicate within-subjects declinations of mental health. What we do have is an indication that participants later in their academic study report poorer mental health. We need to explore this finding further, because if it is the case that academic progress is in some way a risk factor for mental health, then we need to first figure out why and how, and second we need to develop appropriate and ongoing support for those who are engaged in university study.

Data also indicated that participants in a later year of study reported lower S-R generation beliefs, and higher C-M change beliefs, relative to undergraduate study years (when controlling for age). This finding might indicate three things. First, it could be that progression through study years encourages students to adopt more adaptive emotion beliefs through experiential learning. That is, by facing a multitude of challenges associated with continued academic study, students come to understand how best they can regulate their emotions, thus are more likely to endorse C-M change beliefs and relinquish S-R generation beliefs. Second, and in somewhat the other direction causally, it could be that students with more adaptive emotion beliefs are more able and willing to undertake continued study, in part because they can regulate their emotions in times of challenge. But again, these reasonings are mere postulation because we do not have longitudinal data concerning the CMBQ from which we can draw cause-effect conclusions. In addition, whilst some temporal research does indicate that appraisal tendencies might change during university study [e.g., 20, 21], other research indicates no change [e.g., 18, 19]. Third, perhaps level of education is an important antecedent factor for emotion beliefs, such that higher levels of education might be conducive to more adaptive beliefs about emotions. Some research indicates that a higher level of education is positively associated with more adaptive emotion regulation tendencies [e.g., 69, 70], and greater reappraisal tendencies are associated with better academic performance [71]. But on the contrary, data elsewhere indicate a disconnect between reappraisal and level of education [72]. Thus, it might be the case that education level is important for emotion beliefs and emotion regulation more broadly, but researchers are required to take a targeted approach to this question rather than merely controlling for level of education in their analyses as a matter of course.

The present study has some strengths such as the large sample size for the tests conducted, and the rigour with which we approach the CMBQ validity testing prior to main analyses. But the results of the current study should be considered against the backdrop of several limitations. First, this study is cross-sectional, and thus cause-effect conclusions cannot be drawn. To more fully test C-M change and S-R generation beliefs, experimental research should be conducted where these beliefs are manipulated to assess whether the effects of holding either belief predicts differential cognitive reappraisal attempts and subsequent acute emotion reactivity. Researchers could also examine how holding C-M change and S-R generation beliefs may predispose participants to emotion reactivity in response to real stimuli, be it in the laboratory, or in the field. More broadly, researchers could collect more objective emotion reactivity data such as cardiovascular [e.g., 73] and neuroimaging [e.g., 9] indicators. In addition, the results of the current study are specific to a U.K. undergraduate population, and thus generalisability across populations cannot be proffered.

In addition, there are multiple factors that could have been included in our data collection and analyses that are potentially important for emotion beliefs, regulation tendencies, and emotion reactivity. For example, although in the current study the aim was to test the validity of the S-R generation and C-M change concepts in students specifically, participating students may have a number of roles that are pertinent to their emotional experiences. For example, they may be employed in part-time work and are required to, or choose to, balance their studies with work commitments. At the very least, working status should be accounted for in future research concerning the CMBQ, alongside a myriad of socioeconomic factors [e.g., 74, 75] to help us form a more sophisticated and comprehensive picture of factors that can inform emotional experiences. Furthermore, future research could conduct factor analyses on the CMBQ that is stratified across study level. In our data, we found that study level was important for Mean CMBQ scores and for the associations between CMBQ scores and the outcomes, but our data were not suitable (e.g., very low N for doctoral students) for CFA at each study level. It would be useful to know whether and what extent the CMBQ is factorially valid across all levels of study when analysed separately.

There are some potential practical implications of the present study for students, and for those working with students. In line with second-wave CBTs [76, 77], students could be encouraged to recognise the role their beliefs play in their emotions. Further, students could be encouraged to adopt and strengthen C-M change beliefs, whilst weakening S-R generation beliefs, with a view to more volitionally regulate their emotions via cognitive reappraisal. That is, students can exercise some control over their thoughts (although it is taxing) [78], and in turn, can exercise some control over their emotions. This suggestion is in part informed by the results of the current study, but it also a cornerstone of prominent CBTs, especially rational emotive behaviour theory (REBT) [13].

It is also important to outline how the findings of the present study should *not* be used, especially if the results are misinterpreted. The findings here, and those of Turner et al. [32], do not legitimise victim-blaming. That is, it is not that students are to blame for experiencing emotions or for facing emotionally evocative situations. Rather, in the face of a stimulus, students can be encouraged to adopt thoughts and beliefs that make it more likely for them to effectively regulate their emotions. In line with second-wave CBT theory and practice, we suggest that in believing that emotion is cognitively mediated (high C-M change and low S-R generation beliefs), one is more able to regulate one’s emotions.

## Conclusions

In this study we conducted important validity tests concerning the CMBQ in a sample of undergraduate students for the first time. The correlated two-factor structure of the CMBQ was confirmed, and there was evidence of convergent validity, and partial evidence for concurrent validity. A CM-SR discrepancy score, which accounts for both S-R generation and C-M change beliefs, appeared to provide a promising variable when associated with emotion reactivity and positive mental health. However, additional research is required to examine cause-effect implications of S-R generation and C-M change beliefs, and to explore how S-R generation and C-M change beliefs interact to predict emotion reactivity.

## Supporting information

S1 File Study data. (SAV)
